# The association between frailty and incidence of dementia in Beijing: findings from 10/66 dementia research group population-based cohort study

**DOI:** 10.1186/s12877-020-01539-2

**Published:** 2020-04-15

**Authors:** Minghui Li, Yueqin Huang, Zhaorui Liu, Rui Shen, Hongguang Chen, Chao Ma, Tingting Zhang, Shuran Li, Martin Prince

**Affiliations:** 1 Peking University Sixth Hospital, Peking University Institute of Mental Health, National Health Committee Key Laboratory of Mental Health (Peking University), National Clinical Research Center for Mental Disorders (Peking University Sixth Hospital), Beijing, China; 2 Global Health Institute, King’s College London, London, UK

**Keywords:** Ageing, Frailty, Dementia, Follow-up, Cumulative incidence

## Abstract

**Background:**

The relationship between frailty and dementia is unclear and there are very few population-based studies regarding this issue in China. The purpose of this study is to estimate the association between frailty and incident dementia in China, and to explore different effects of frailty established by three definitions of frailty on dementia incidence.

**Methods:**

A five-year prospective cohort study was carried out in 2022 participants aged 65 years and over in urban and rural sites in Beijing, China. The participants were interviewed by trained community primary health care workers from 2004 to 2009. Frailty was defined using modified Fried frailty phenotype, physical frailty definition, and multidimensional frailty definition. Dementia was diagnosed using the 10/66 dementia criterion for calculating cumulative incidence. Both competing risk regression models and Cox proportional hazards models were applied to examine the associations between frailty at baseline and five-year cumulative incidence of dementia.

**Results:**

At the end of follow-up the five-year cumulative incidence rates of dementia with frailty and without frailty defined by the modified Fried frailty were 21.0% and 9.6%, those defined by the physical frailty were 19.9% and 9.0%, and those defined by the multidimensional frailty were 22.8% and 8.9%, respectively. Compared with non-frail participants, frail people had a higher risk of incident dementia using multidimensional frailty definition after adjusting covariates based on competing risk regression model (HR = 1.47, 95% CI 1.01~2.17) and Cox proportional hazards model (HR = 1.56, 95% CI 1.07~2.26). The association between frailty and incident dementia was statistically significant in participants in the upper three quartiles of age (aged 68 years and over) using the multidimensional frailty definition based on the competing risk regression model (HR = 1.61, 95% CI 1.06~2.43) and Cox proportional hazard model (HR = 1.76, 95% CI 1.19~2.61).

**Conclusions:**

Multidimensional frailty may play an inherent role in incident dementia, especially in the people aged over 68, which is significant for distinguishing high risk people and determining secondary prevention strategies for dementia patients.

## Background

The number of people with dementia worldwide is rising within the ageing population, which is a severe issue in all countries. The World Health Organization (WHO) reports that there are almost 50 million people currently living with dementia, and nearly 10 million new cases each year [1], with the highest growth area being in China and some other developing nations [2]. According to the China Mental Health Survey, the prevalence of dementia for the population aged 65 and over is 5.6% diagnosed by the Diagnostic and Statistical Manual of Mental Disorders (DSM)-IV [3–5]. Moreover, the prevalence is higher if using the 10/66 diagnostic package [6], which is more sensitive for detecting the early stages of dementia.

Because of the limited availability of therapeutic medications for dementia, it is particularly important to identify its risk factors, which can help policy makers distinguish and intervene in high-risk cases. Therefore, considerable attention to risk factors for dementia has been sustained over the last two decades. Age, smoking, hypertension, high cholesterol, and apolipoprotein E are considered likely risk factors for dementia [7]. In addition, there is a consensus that the risk factors of incident dementia occurrence likely cluster with lifestyles, chronic diseases, and physical impairment [8–11]. Moreover, in a meta-analysis on dementia, a relationship was found between exposure to increased factor load and subsequent poorer cognitive function or dementia [12].

Some researchers have suggested integrating risk factors into definition of frailty, which is a comprehensive indicator for evaluating its effects on dementia incidence [13]. Frailty is an age-related syndrome with the main features of impaired multisystem physiological reserves and decreased resistance to substantial stressors [14].

It was estimated that frailty increases the likelihood of future cognitive decline and that cognitive impairment elevates the risk of frailty. However, the mechanism of the potential association between frailty and incident dementia is unclear. It has been shown that physical frailty increases the risk of diabetes mellitus and cardiovascular disease [15, 16]. Moreover, a clinical-pathologic study found physical frailty to be associated with dementia pathology [17–19], and alterations in hippocampal synaptic function, neuronal membrane properties, and axonal trajectories, which might lead to dementia have been reported for people with frailty [20–22].

Preliminary studies on frailty in China have focused on the association between frailty and death. Dupre reported the relationship between frailty, type of death and suffering death, using the frailty index comprising 39 deficits [23]. The most frequently used frailty assessment methods in China are the Fried phenotype and the frailty index, which are similar to those in other countries. For community-dwelling people aged 65 or over in China, the prevalence of frailty is 5.9%–17.4% [24], close to that in developed countries. Nonetheless, very few studies on the relationship between frailty and dementia have been carried out in China. In addition, the prevalence of frailty varied in different studies due to the assessment method employed, and previous studies have shown that the variables selected for assessment methods differwith regard to study aim.

## Methods

### Objective

The objectives of this study were to estimate the potential association between frailty and incident dementia in China and to explore different effects of frailty established by three definitions of frailty on dementia incidence.

### Design and participants

This study is one part of the 10/66 dementia research group population-based studies, which were carried out in people aged 65 years and over living in 11 catchment areas (China, Cuba, Dominican Republic, India, Mexico, Peru, Puerto Rico, and Venezuela). The present study only included data for China. The participants included were from the baseline survey conducted by trained psychiatrists from January 2004 to April 2005, and all residents aged 65 years and over living in the catchment areas of Xicheng and Daxing were enrolled. Moreover, full follow-up was performed four to five years after the baseline. For deceased participants, the international standard verbal autopsy questionnaire was used to obtain information on death and probable causes or causes of death [25]. Comprehensive details of the study population have been published elsewhere [26].

### Measurements

#### Dementia

The method used for evaluating dementia was the 10/66 dementia diagnosis adjusted education, which was developed by the 10/66 dementia research group; it has been widely used in previous studies. The information for establishing diagnoses included the Community Screening Instrument for Dementia (CSI-D), the Geriatric Mental State (GMS), and a structured neurological examination [27, 28]. The CSI-D was used to collect information on cognitive and functional decline. The GMS was applied to identify organic brain syndrome (probable dementia), depression, anxiety and psychosis. The structured neurological examination assessed whether the participants had lateralizing signs, Parkinsonism, ataxia, apraxia and primitive reflexes. More details about the package have been published elsewhere [29].

#### Frailty

In this study, frailty was defined using three types of definitions. The first definition was the modified Fried frailty phenotype, which included four variables: exhaustion, weight loss, slow walking speed, and low energy expenditure. In the 10/66 cohort study, handgrip strength was omitted. Participants were identified as frail if they exhibited two or more of the four variables [30].

Exhaustion was assessed by Q48.1 in the GMS questionnaire. Participants who self-reported feeling worn out or exhausted were considered potentially frail. Weight loss status was evaluated by the question “have you lost 10 lbs (4.5 kilograms) in weight during the past three months?” Slow walking speed was defined by a walking timing test (walk 5 m at usual speed, turn, and return to the starting point), applying the cut-off point of 16 s or longer to complete the task to indicate frailty.

The second definition was generated using the Rockwood frailty index construction [31]. Based on the modified Fried frailty phenotype, nine physical impairment items were added to describe frailty constituting physical frailty. The physical impairment items included: finger movement disorder, decreased muscular tension, abnormal tendon reflex, eyesight problems, hearing difficulty or deafness, dysdiadochokinesis, waist-hip ratio (≥ 0.85 in female or ≥ 0.90 in male regarded as frail), sleeping disorders, and slow action. The participant’s frailty index was equal to the number of unhealthy items divided by the total number of items. Frailty was defined as having a physical frailty index (PFI) greater than or equal to 0.25 [32].

The third definition was the multidimensional frailty index (MFI), which added the mental status and social relations of the elderly into the PFI. Items added to the MFI included impairment of general cognitive ability, delayed recall problems, lack of concentration, lack of interest, delayed recall, depressive emotions, loneliness, and insufficient social support.

#### Other associated assessments

Other potential risk factors were selected from prior studies, including sociodemographic factors, physical impairments, behaviours and lifestyles.

Sociodemographic factors were collected through interviews, including age, gender, marital status (unmarried, married/co-habiting, widowed/divorced/separated), and education level (none, minimal/completed primary, completed secondary/metric, completed tertiary/college/further education).

The participants or their proxies reported a history of the following conditions: arthritis/rheumatism, persistent cough, breathlessness/difficulty breathing/asthma, hypertension, cardiopathy/angina, gastrointestinal problems, paralysis/weakness/loss of one leg or arm, skin disorders, fainting or blackouts, and diabetes mellitus [33]. Those participants with three or more physical impairments were defined as having physical multimorbidity. Physical multimorbidity reflected the most common physical impairments that interfere with older people’s daily activities.

In the study, alcohol use status (non-drinking, temperance, drinking), smoking status (no smoking, smoking cessation, smoking), and walking distance per week (less than 3 km, more than or equal to 3 km) were collected at the same time.

#### Statistical analysis

SAS version 9.4 was used for all analyses. Participants who did not have dementia at baseline constituted the cohort. First, the participants’ baseline characteristics for each catchment area, including age, gender, education level, marital status, smoking status, and alcohol use status, were collected. Moreover, differences in variables between rural (Daxing) and urban (Xicheng) participants’ baselines were compared by percentages and *Pearson’s chi-square test* and the difference in age between the two settings was compared by means and the *t-test.*

For each frailty definition, five-year cumulative incidence rates were calculated and competing risk regression model and Cox proportional hazards model were used for analyses. Competing risk regression models were employed according to the methods of Fine and Gary, taking into account death as the competing risk event of incident dementia. Cox proportional hazards regression models were used to examine the relationship between frailty and mortality in the sample population while controlling for selected covariates. After the unadjusted model (model 1) was determined, further adjustments for age, gender, and education level (model 2) were included. Model 3 included all components of model 1 and model 2, as well as physical multimorbidity, walking distance per week, smoking status and alcohol use. The hazard ratios (HRs) with 95% confidence intervals (CIs) of incident dementia by frailty are reported.

Regarding Doraiswamy and Bieber’s research about dementia assessment scales [34], separate analyses were dichotomized into two groups, the lowest quartile and upper three quartiles of age at baseline. The cut-off point was 68 years old. To explore whether age modifies the association between frailty and incident dementia, an interaction term between frailty and age at baseline was derived, controlling for gender, education level, physical multimorbidity and alcohol use.

## Results

### Sample characteristics

The sample characteristics of the analyses are displayed in Table 1. A total of 2087 participants were assessed at baseline; 65 participants who had dementia at baseline were excluded. The mean follow-up period was 4.92 years, with a standard deviation (SD) of 0.99 years. Among the 2022 participants at baseline, 386 died during the follow-up period, with a mortality of 19.1%. The quartiles of age were 68, 72 and 76 among all participants. Demographic ageing was more prevalent in the urban site than that in the rural site (*t* = 5.499, *p* < 0.001). The means of age in the rural and urban sites were 72.05 and 73.47 years, respectively (Table 1). There were 609 females (56.6%) in the urban site and 521 (55.1%) in the rural site. There was no significant difference by gender between the urban and rural sites. Education levels in the urban site were higher than those in the rural site, with “Minimal/completed primary” being the majority (*N* = 423; 39.3%) among the urban participants. Regarding marital status, 780 (72.5%) participants in the urban site and 562 (59.4%) in the rural site were married. The prevalence rates of alcohol use in the rural and urban sites were 16.3% and 3.4%, respectively. The prevalence rate of smoking in the rural site was 30.4%, which was higher than that in the urban site (17.0%).

**Table 1:** Sample Characteristics at Baseline

Demographic Variables	Total *N* = 2022		Urban *N* = 977		Rural *N* = 948		*t/χ*^*2*^	*P*
	N	%	N	%	N	%		
Gender							0.5	0.49
Female	1130	55.9	609	56.6	521	55.1		
Male	892	44.1	467	43.4	425	44.9		
Age							34.7	<.001
65–69	686	33.9	309	28.7	377	39.9		
70–74	635	31.4	350	32.5	285	30.1		
75–79	419	20.7	236	21.9	183	19.3		
80–84	182	9.0	118	11.0	64	6.8		
85+	100	5.0	63	5.9	37	3.9		
Mean	72.80		73.47		72.05		5.499^*^	<.001
25th percentile	68		69		68			
50th percentile	72		73		71			
75th percentile	76		77		75			
Education level							473.6	< 0.001
None	748	37.0	207	19.2	541	57.2		
Minimal/completed primary	781	38.6	423	39.3	358	37.8		
Completed secondary/metric	358	17.7	316	29.4	42	4.4		
Completed tertiary/college/further education	135	6.7	130	12.1	5	0.5		
Marital status							49.0	<.001
Unmarried	25	1.2	3	0.3	22	2.3		
Married/co-habiting	1342	66.4	780	72.5	562	59.4		
Widowed/divorced/separated	655	32.4	293	27.2	362	38.3		
Alcohol use status							106.8	<.001
No drinking	1803	89.2	1031	95.8	772	81.6		
Temperance	29	1.4	9	0.8	20	2.1		
Drinking	190	9.4	36	3.4	154	16.3		
Smoking status							63.4	<.001
No smoking	1449	71.7	817	75.9	632	66.8		
Smoking cessation	102	5.0	76	7.1	26	2.8		
Smoking	471	23.3	183	17.0	288	30.4		

*N* Number of participants

^*^ The difference of age between the two sites was compared by means and *t-test*

### Association between frailty and incident dementia

Table 2 displays the number and percentage of participants deemed frail at baseline and the five-year cumulative incidence of dementia at the end of follow-up. The five-year cumulative incidence of dementia in the participants exhibiting baseline frailty was twice as high as that in the participants deemed not to be frail at baseline. There were 105 modified Fried frail participants at baseline, and 21.0% of them developed dementia by the end of follow-up. However, only 9.6% of non-modified Fried frail participants developed dementia. There were 206 physically frail participants and 197 multidimensionally frail participants at baseline. The five-year cumulative incidences of dementia were 19.9% and 22.8%, respectively.

**Table 2:** Frailty of Prevalence at Baseline and Incidence of Dementia at the End of Follow-up

Model	Frailty prevalence at baseline		5-year cumulative incidence of dementia (%, 95% CI)	*χ*^*2*^	*P*
	N	Prevalence (%)			
Modified Fried Frailty				14.2	<.001
No frailty	1917	94.8	9.6(8.2,10.9)		
Frailty	105	5.2	21.0(13.2,28.7)		
Physical Frailty				24.0	<.001
No frailty	1816	89.8	9.0(7.7,10.4)		
Frailty	206	10.2	19.9(14.2,25.4)		
Multidimensional Frailty				38.7	<.001
No frailty	1825	90.3	8.9(7.5,10.1)		
Frailty	197	9.7	22.8(17.0,28.7)		

*N* Number of participants; *CI* Confidence interval

Table 3 reports the HRs based on competing risk regression models and Cox proportional hazards models. Of the modified Fried frailty phenotype unadjusted models, the HRs generated from the competing risk model and Cox proportional hazards model were 1.71 (95% CI: 1.09~2.68) and 1.78 (95% CI: 1.14~2.79) respectively. When adjusting for age, gender and education level, no significant differences in the association between frailty and incident dementia were observed in either model. Of the physical frailty unadjusted models, the HRs based on the competing risk model and the Cox proportional hazards model were 1.78 (95% CI: 1.25~2.54) and 1.78 (95% CI: 1.25~2.53), respectively. Of the multidimensional frailty models, when adjusting for age, gender, education level, physical multimorbidity, walking distance per week, smoking status, and alcohol use, the HRs based on the competing risk model and Cox proportional hazards model were 1.47 (95% CI: 1.01~2.17) and 1.56 (95% CI, 1.07~2.26), respectively.

**Table 3:** Competing Risk Model and Cox Proportional Hazards Model of Frailty and Incident Dementia by three Frailty definitions

Model^*^	Modified Fried Frailty Phenotype		Physical Frailty		Multidimensional Frailty	
	Competing risk model*HR* (95% CI)	Cox proportional hazards model *HR* (95% CI)	Competing risk model *HR* (95% CI)	Cox proportional hazards model*HR* (95% CI)	Competing risk model*HR* (95% CI)	Cox proportional hazards model*HR* (95% CI)
Model 1						
No Frailty	1.00	1.00	1.00	1.00	1.00	1.00
Frailty	1.71(1.09, 2.68)	1.78(1.14, 2.79)	1.78(1.25, 2.54)	1.78(1.25, 2.53)	2.14(1.53, 3.00)	2.15(1.53, 3.02)
Model 2						
No Frailty	1.00	1.00	1.00	1.00	1.00	1.00
Frailty	1.25(0.78, 2.01)	1.33(0.84, 2.09)	1.37(0.93, 2.01)	1.32(0.92, 1.89)	1.65(1.16, 2.34)	1.66(1.17, 2.33)
Model 3						
No Frailty	1.00	1.00	1.00	1.00	1.00	1.00
Frailty	1.14(0.69, 1.87)	1.14(0.70, 1.84)	1.16(0.78, 1.73)	1.24(0.83, 1.85)	1.47(1.01, 2.17)	1.56(1.07, 2.26)

*HR* Hazard Ratio; *CI* Confidence interval

*Model 1: unadjusted; Model 2: adjusted age, gender, education level; Model 3: adjusted age, gender, education level, physical multimorbidity, walking distance, smoking status and alcohol use

The HRs for the competing risk models and the Cox proportional hazards models of three definitions of frailty in two age groups, adjusted gender, education level, physical multimorbidity, walking distance per week, smoking status and alcohol use are provided in Table 4; the boundary was 68 years old as the lowest quartile age [34]. For participants in the upper three quartiles of age, the HRs for multidimensional frailty based on the competing risk model and Cox proportional hazards model were 1.61 (95% CI: 1.06~2.43) and 1.76 (95% CI: 1.19~2.61), respectively.

**Table 4:** Competing Risk Models and Cox Proportional Hazard Models of Frailty and Incident Dementia in Two Age Groups^*^

Frailty definition	Lower quartile of age(*N* = 538)		Upper three quartiles of age(*N* = 1484)	
	Competing risk model *HR* (95% CI)	Cox proportional hazards model *HR* (95%CI)	Competing risk model *HR* (95% CI)	Cox proportional hazards model *HR* (95%CI)
Modified Fried Frailty Phenotype				
No frailty	1.00	1.00	1.00	1.00
Frailty	0.89(0.23, 3.50)	0.96(0.16, 5.61)	1.25(0.75, 2.08)	1.28(0.77, 2.15)
Physical Frailty				
No frailty	1.00	1.00	1.00	1.00
Frailty	1.94(0.481, 7.80)	2.13(0.48, 9.37)	1.28(0.83, 1.96)	1.38(0.92, 2.08)
Multidimensional Frailty				
No frailty	1.00	1.00	1.00	1.00
Frailty	1.52 0.47, 4.86)	1.643(0.46, 5.84)	1.61 (1.06, 2.43)	1.76(1.19, 2.61)

*N* Number of participants; *HR* Hazard Ratio; *CI* Confidence interval

*Risk of incident dementia is adjusted age, gender, education level, physical multimorbidity, walking distance per week, smoking status alcohol use

## Discussion

The study is one part of the series of studies on dementia by the 10/66 International Research Group. To date, very few studies have explored the relationship between frailty and dementia. Based on three different definitions of frailty, we explored the probable influence of frailty on the incidence of dementia in elderly people in a Beijing sample. The results indicated that the participants with multidimensional frailty had a higher risk than did non-frail participants and that the size of the effect varied across different age groups. This finding is consistent with previous studies showing that frailty is a risk factor for dementia [35]. The uniqueness of this study is the fact that the competing risk of death was considered when exploring the influence of frailty on incident dementia in China [36], which can result in accurate HRs, despite a limitation to using those experiences directly because of the difference between China and other Western countries. Although the results of the three definitions differ slightly, frailty can generally increase the hazard of incident dementia.

The modified Fried frailty phenotype, which was used in previous frailty studies by 10/66 collaboration group, includes four physical variables, and this definition has been confirmed as a good predictor for dependence and mortality in older adults [30]. Regardless, the results did not prove the association between the modified Fried frailty phenotype and dementia after adjusting for age, gender and education level. In other words, the modified Fried frailty phenotype could not accurately predict the risk of incident dementia in this Chinese sample. To further evaluate the association between physical frailty and incident dementia, nine additional physical variables, including finger movement disorder, decreased muscular tension, abnormal tendon reflex, eyesight problems, hearing difficulty or deafness, dysdiadochokinesis, waist-hip ratio, sleeping disorders, and slow action, were added to the modified Fried frailty phenotype forming the PFI, but the result was similar to that of the modified Fried frailty phenotype. The association between PFI and incident dementia was only found in the unadjusted model. Previous population-based studies have reported that physical frailty might be associated with vascular dementia, which accounts for 15%~ 20% of all-cause dementia [37]. As only all-cause dementia was considered in this study, the association between it and physical frailty was not as expected. It is assumed that physical frailty plays different roles in predicting the incidence of different types of dementia. To explore this question more deeply, two age-group analyses were conducted to assess the relationship between frailty and incident dementia.

The mental status and social relations of elderly individuals were added, including impairment of general cognitive ability, delayed recall problems, lack of concentration, lack of interest, delayed recall, depressive emotions, loneliness, and insufficient social support, to the definition of multidimensional frailty. The results showed that multidimensional frailty is an independent predictor for incident dementia and associated with a 47% increased hazard in the competing risk model after adjusting for age, gender, education level, physical multimorbidity, walking distance per week, alcohol use and smoking status. Most previous studies on frailty have only focused on the physical dimension [16, 17, 38], though there is some evidence showing a relationship between mental status and social relations and incident dementia. In a Japanese dementia study, Shimada interviewed 4570 community-dwelling elderly persons and found that inclusion of a cognitive dimension in the frailty assessment improved the predictive accuracy [39]. This finding suggests that the identification of high-risk populations of dementia should not be limited to one dimension. A better identification should include multidimensional risk factors for identifying high-risk populations and providing advanced intervention. The report of the Lancet International Commission recommended that dementia prevention include more childhood education, exercise, and management of hearing loss and depression [40]. In addition, Rogers derived a frailty index comprising 47 health factors and found that frailty should be considered alongside cognitive function when assessing risk factors of dementia [41]. Furthermore, it has been shown conclusively that cognitive impairment is a potential component of the secondary prevention of dementia [42]. There are several possible explanations for this result. First, frailty is a condition that is characterized by a reduction in the homeostatic reserves of the individual, which entails an increased vulnerability to endogenous and exogenous stressors [43]. Hence, frail people face more challenges in adapting to significant threats that may occur in that individual’s life. In other words, frail people’s resilience may decline, which will increase the likelihood of dementia. Second, frailty and dementia might have a similar aetiological pathway because both are related to cognitive decline [41]. The aetiology of frailty is multidimensional, including nutrition, physical activity, coping, social support and other potential causes [44, 45]. Inflammation, oxidative stress and cortical atrophy are three factors that play essential roles in the development of both dementia and frailty [46]. Previous studies reported that in a population of cognitively declining patients, frailty is associated with the presence, degree, and localization of cerebral atrophy. For every 1% increase in FI, the probability of cortical atrophy increased by 2% [43].

In a long-term follow-up cohort study, participants may become deceased during the follow-up period, especially those who are frail. In this study, the mortality of the participants was almost one-fifth, and these censored data can lead to bias. Therefore, competing risk models were applied to analyse death as a competing event to dementia, which decreased the bias of non-dementia premature death in the association of frailty and incident dementia. Table 3 shows that multidimensional frailty was more highly associated with incident dementia after controlling for age, gender, education level, physical multimorbidity, walking distance per week, smoking status, and alcohol use. The HRs of competing risk hazard models were lower than those of the Cox proportional hazards model. The results of this study suggest that the Cox proportional hazards model might overestimate the risk of incident dementia but that the competing risk hazard model is able to calculate unbiased and accurate outcomes.

According to the data in Table 4, frailty was not associated with incident dementia in the lowest quartile age group of the participants at baseline. In contrast, the other group had a significantly higher risk of incident dementia if they met the definition of multidimensional frailty at baseline. Thus, there was a positive association between age and multidimensional frailty. The gradual decline in physiological reserve with ageing accelerates faster in frail people than in non-frail people [47]. This observation is also in-line with other studies, showing that the risk of incident dementia increases with decreasing physiological reserve in elderly individuals [48].

There are two limitations in this study. First, grip strength information was not collected at the baseline, so the models of this study did not analyse grip strength as a variable of frailty. But previous studies of 10/66 group have suggested that the link between grip strength and adverse health conditions weakens when other frailty indicators and confounding factors are accounted for [49], although grip strength was one of the five indicators of Fried frailty phenotype. Therefore, this study was consistent with former 10/66 dementia study [30]. Second, this cohort study was not nationally representative, so the external validity of prevalence and cumulative incidence was not as good as the internal validity. However, this study did focus on exploring the association between frailty and dementia, rather than on the prevalence of dementia in the national population.

In the future, additional studies are needed to confirm the associations between subtypes of dementia and frailty to determine potential causes of dementia to prevent elderly people from developing the condition. Other important comorbidities, such as hyperlipidaemia, stroke, or cancers, require further analyses. Moreover, to explain the mechanism between frailty and dementia in dementia studies, biomarkers should be considered vital mediators for predicting the condition. Future cohort studies should take into consideration psycho-social and biological factors to build a more accurate prediction model.

## Conclusions

Frailty, including physical and cognitive dimensions, plays an inherent role in incident dementia, being more significant in elderly people. Compared to physical frailty, multidimensional frailty assessment formed by physical, mental status and social relations, can better explain the dynamic association between frailty and incident dementia. The present findings are significant for distinguishing high-risk older persons and may provide an opportunity for intervention to reduce the impacts of dementia on personal and social well-being.

## Data Availability

The data underlying this study are restricted, as participants did not consent to sharing their information publicly. Data underlying the results presented in the study are available from the 10/66 Dementia Research Group public data archive for researchers who meet the criteria for access to confidential data. Information on procedures to request access is available at https://www.alz.co.uk/1066/1066_public_archive_baseline.php, or by contacting dementiaresearchgroup1066@kcl.ac.uk

## Abbreviations

CI: Confidence interval
CSI-D: Community Screening Instrument for Dementia
DSM: Diagnostic and Statistical Manual of Mental Disorders
GMS: Geriatric Mental State
HRs: Hazard ratios
MFI: Multidimensional Frailty Index
PFI: Physical Frailty Index
SD: Standard deviation
WHO: World Health Organization
